# Correlations in Ion Channel mRNA in Rhythmically Active Neurons

**DOI:** 10.1371/journal.pone.0006742

**Published:** 2009-08-25

**Authors:** Anne-Elise Tobin, Nelson D. Cruz-Bermúdez, Eve Marder, David J. Schulz

**Affiliations:** 1 Department of Biology, Brandeis University, Waltham, Massachusetts, United States of America; 2 Department of Biological Sciences, University of Missouri, Columbia, Missouri, United States of America; Mount Sinai School of Medicine, United States of America

## Abstract

**Background:**

To what extent do identified neurons from different animals vary in their expression of ion channel genes? In neurons of the same type, is ion channel expression highly variable and/or is there any relationship between ion channel expression that is conserved?

**Methodology/Principal Findings:**

To address these questions we measured ion channel mRNA in large cells (LCs) of the crab cardiac ganglion. We cloned a calcium channel, *caco*, and a potassium channel, *shaker*. Using single-cell quantitative PCR, we measured levels of mRNA for these and 6 other different ion channels in cardiac ganglion LCs. Across the population of LCs we measured 3–9 fold ranges of mRNA levels, and we found correlations in the expression of many pairs of conductances

**Conclusions/Significance:**

In previous measurements from the crab stomatogastric ganglion (STG), ion channel expression was variable, but many pairs of channels had correlated expression. However, each STG cell type had a unique combination of ion channel correlations. Our findings from the crab cardiac ganglion are similar, but the correlations in the LCs are different from those in STG neurons, supporting the idea that such correlations could be markers of cell identity or activity.

## Introduction

Increasing theoretical and experimental evidence shows that, within a neuron type, the abundance of the ion channels that shape neuronal activity can vary significantly across individual neurons without affecting their ability to produce remarkably similar activity [1]–[10]. How can activity be relatively stable despite variation in the conductances that give rise to that activity? One possibility is that compensatory changes occur in multiple conductances to preserve activity.

In neurons of the rhythmically active stomatogastric ganglion (STG) in the crab (*Cancer borealis*), single-cell measurements of mRNA transcripts were both variable and correlated with voltage-clamp measurements of conductance [6], indicating that variability is intrinsic and is present at the level of gene transcription and membrane conductance. Similar results were found in voltage-clamp (I_A_) and RT-PCR (Kv4.3L) measurements from individual dopaminergic striatal neurons [11]. Moreover, in the STG, while transcript levels varied up to five-fold across cells, a linear relationship between certain pairs of ion channel mRNA levels was observed [6], [12]. Such a relationship among ion channel mRNA levels may result from homeostatic mechanisms designed to maintain cell activity despite perturbations [13]–[20]. The ion channel correlations in STG neurons were cell-type specific, suggesting that each identified neuron type might display a characteristic set of correlations, and that these correlations might be a marker for cell identity or activity [12]. Similarly, in the dopaminergic midbrain system of the mouse, the ratios of expression of several dopaminergic marker genes were cell-type specific, corresponding to the neurons pattern of projection [21].

**Figure 1:**
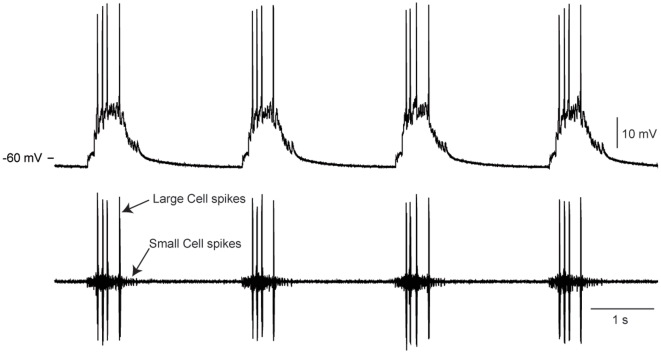
Spontaneous activity of the cardiac ganglion in *C. borealis*. Top: intracellular recording from the axon of a LC. Bottom: simultaneous extracellular recording from the cardiac ganglion trunk, showing small cell (small units) and large cell (LC) (tall units) bursts of action potentials.

In this study we extended the work from the crab STG to another rhythmically active network, the cardiac ganglion, in the same species, *Cancer borealis*. The nine-neuron cardiac ganglion consists of five large cell (LC) motor neurons and four small pacemaker cells, all of which are electrically coupled and burst synchronously to drive the contraction of the heart muscle [22]–[26] (Fig. 1). Studies of LCs in the crab *Portunus sanguinolentus* have shown that an inward calcium current, *I_Ca_*, is responsible for regenerative depolarization in response to injected current [27]–[31]. Three outward potassium currents have been found in LCs [28], [29]: an early outward current *I_A_*, a delayed outward current *I_Kd_*, and a calcium-dependent potassium current *I_KCa_*. Action potentials are abolished by application of TTX and assumed to be generated by a fast sodium current, *I_Na_* [27]–[31]. The *C. borealis* cardiac ganglion responds generally similarly to neuromodulators as do the cardiac ganglia of other species, suggesting the basic structure and function of the cardiac ganglion is similar across decapod crustaceans [32].

To expand our understanding of cell-specific patterns of ion channel expression, we set out to determine whether neurons of another network would show the same or different correlations in channel gene expression as those seen in the STG. Similar to STG neurons, the LCs of the cardiac ganglion have a large enough cytoplasmic volume to enable single-cell measurements of multiple mRNA transcripts. Taking advantage of this, we measured the mRNA levels for eight different ion channels in individual LCs. As in STG neurons, we found clear correlations in the mRNA levels of expression of some of these genes. The combination of channel correlations in the LCs was different from any of the unique combination of correlations in the STG neurons, supporting the idea that these correlations are markers of cell identity or activity.

## Results

A number of ion channel genes from *C. borealis* have been previously cloned and sequenced [6], [12]. These include: *para* (I_Na_), *shal* (I_A_), *shab* and *shaw* (I_Kd_), *BKKCa* (I_KCa_) and *IH* (I_h_). We now add two additional genes to this list, bringing to 8 the number of ion channel genes that we were able to measure in single, identified neurons.

### Cloning of *shaker* and *caco* channels in *C. borealis*

RT-PCRs with degenerate primers and *C. borealis* brain cDNA template produced partial open reading frames for two channel orthologs from the crab: 1016 base pairs of a potassium channel that encodes for a fast transient A-type current, (*shaker*), and 886 base pairs of *cacophony*, (referred to here as *caco*) a voltage-gated calcium channel alpha subunit that influences many characteristics of high-voltage activated calcium currents in *Drosophila* [33]. As revealed by the BLASTX algorithm (NCBI), the Cab-*shaker* sequence shares 89% identity at the amino acid level with *Drosophila melanogaster shaker* sequence (Accession #NP_728123), while the Cab-*cacophony* sequence shares 84% amino acid identity to that of *D. melanogaster cacophony* (NP_001014735).

### Quantification of mRNA levels

Previous current-clamp and voltage-clamp studies from cardiac ganglion LCs in *Portunus sanguinolentus* identified one sodium current, I_Na_, a calcium current with multiple inactivation time constants, I_Ca_, and three potassium currents, I_A_, I_Kd_, and I_KCa_. Similar currents have been measured in STG neurons and the following transcripts likely to be responsible for these currents (due to high sequence similarity with channels characterized in related organisms) have already been characterized: *para* (I_Na_), *shal* (I_A_), *shab* and *shaw* (I_Kd_), *BKKCa* (I_KCa_) and *IH* (I_h_). For this study we also cloned and sequenced two channels previously uncharacterized channels, *shaker* (I_A_) and *caco* (I_Ca_). *Shaker* is well known to carry I_A_ in a closely related species, *Panulirus interruptus* [34], [35]. *Cacophony* is a well characterized voltage-gated calcium channel [33], but as of yet it is not clear what the kinetics of the current carried by this channel are in crustaceans, and whether it corresponds to all or part of I_Ca_. We measured levels of these 8 transcripts in the *C. borealis* cardiac ganglion LC cells.

**Figure 2:**
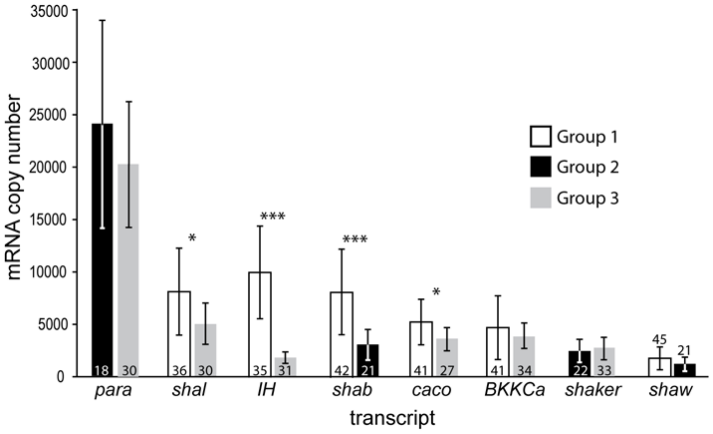
Mean mRNA copy numbers for 8 ion channel gene transcripts in LCs. The LC groups were harvested and processed at different times during the year. The numbers of cells included in each analysis (n) are indicated inside or above standard deviation bars. Asterisks indicate significance differences at p<0.01 (*) or p<0.001 (***) for the same channel between two groups of cells (*t*-test).

Our current analysis capabilities allow for up to 6 channel genes to be quantified from a single neuron. Because ultimately we were interested in comparing expression levels across channel type (Table 1), 3 groups of cells, from which 4 to 6 gene transcripts were quantified concurrently in each LC, were required to account for the 28 possible pairwise comparisons among 8 channel genes. These 3 groups were harvested at different times of the year: Group 1 in August, Group 2 in October and Group 3 in November. The anatomical locations of the 5 LCs vary somewhat within the *C. borealis* cardiac ganglion, but typically 3 LCs reside in the anterior branches of the ganglion, and two in the posterior end of the main trunk. We harvested as many LCs per ganglion as possible, excluding any cells that appeared to be damaged during the dissection. As there is no evidence for electrophysiological differences between the LCs, we pooled the data without regard to anatomical location of each LC, except where indicated. On average, the *para* transcript was most highly expressed, followed, in decreasing order by *shal, IH*, *shab*, *caco BKKCa*, *shaker*, and *shaw* (Fig. 2). For four of the eight channels measured, the mean channel levels differed between batches of LCs. Group 1 neurons had, on average, more, *shal*, *IH*, and *caco* than Group 3, and more *shab* than Group 2. In the two groups where *para* was measured, it was the most abundant channel transcript. The amount of *IH* varied most dramatically between groups, being 5 times higher in Group 1 than in Group 3. As such, *IH* is the second most abundant transcript of those measured in Group 1 (more than double *BKKCa*), but the least abundant transcript of those measured in Group 3 (less than half *BKKCa*). The large standard deviations in Figure 2 reflect the substantial cell to cell variability in the expression levels for each of these channel genes, as was also seen in previous work [6], [12].

**Table 1:** Coefficients of determination (R^2^) for conductance correlations.

transcripts	*BKKCa*	*IH*	*shal*	*shab*	*shaw*	*caco*	*para*
***BKKCa***							
***IH***	**0.40/0.36**						
***shal***	**0.58/0.36**	0.29**/0.52**					
***shab***	0.06	0.18	0.27				
***shaw***	0.01	0.00	0.05	0.20			
***caco***	0.12/**0.50**	0.15/0.33	**0.28/0.46**	***0.83***	0.17		
***para***	**0.40**	*	***0.60***	***0.63***	0.24	***0.69***	
***shaker***	***0.73***	**0.39**	**0.50**	0.16	0.33	**0.44**	***0.61/0.74***

Coefficients of determination (R^2^ values) for linear regression fits of the dependence of mRNA levels of one ion channel versus those of another. **Bold** indicates significant fits (p<0.0014; Bonferroni adjusted) with R^2^ values<0.6; ***bold and italics*** indicates significant fits with R^2^ values> = 0.60. These “strong” correlations represented in bold and italics are plotted in Fig. 5. For pairs that were measured in more than one group, both R^2^ values are presented. *A significant correlation was measured between normalized levels of *para* and *IH* (R^2^ = 0.51), however no significant correlation was measured in the unnormalized data. To avoid the possibility of normalization inducing false correlations, we exclude these data from our analysis.

**Figure 3:**
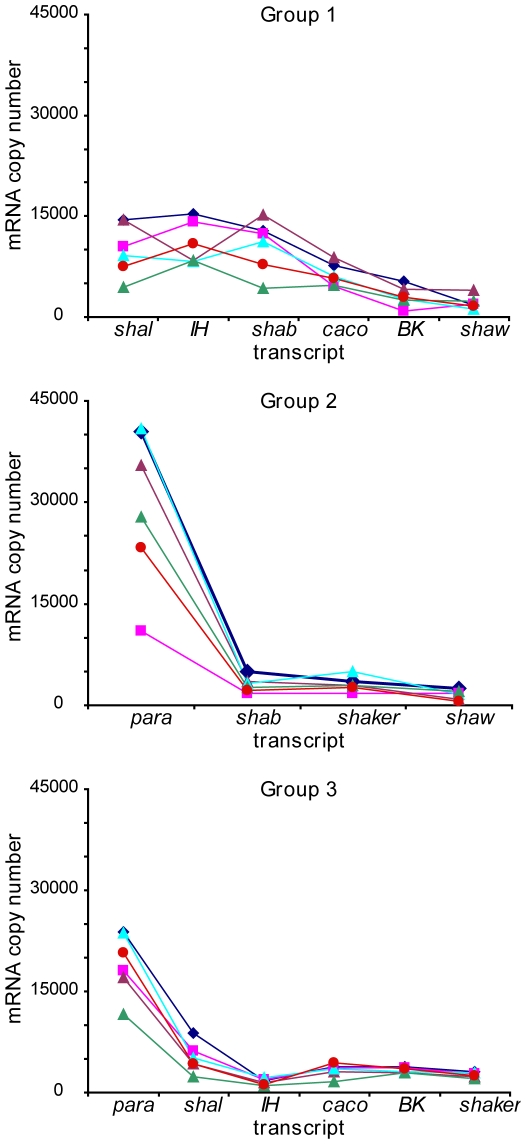
Example ion channel mRNA profiles from 3 groups of LCs. All cells in each group are processed together; these LCs were randomly selected among the cells with measurements for each transcript.

In all cells where it was measured, *para* was the most abundant transcript, and in 84% of cells where it was measured, *shaw* was the least abundant. The expression profile, however, was not completely consistent across cells. The mean number of *para* copies, across all groups, was more than 14 times that of the *shaw* transcript, but the ratio varied considerably across cells, from 9 to 56-fold, as demonstrated in the expression profiles of several example cells (Fig. 3). Additionally, the order of expression abundance varied somewhat amongst the cells within each group and between groups. *Shal* was greater than *IH* in all of the Group 3 cells, but only in one third of the Group 1 cells.

**Figure 4:**
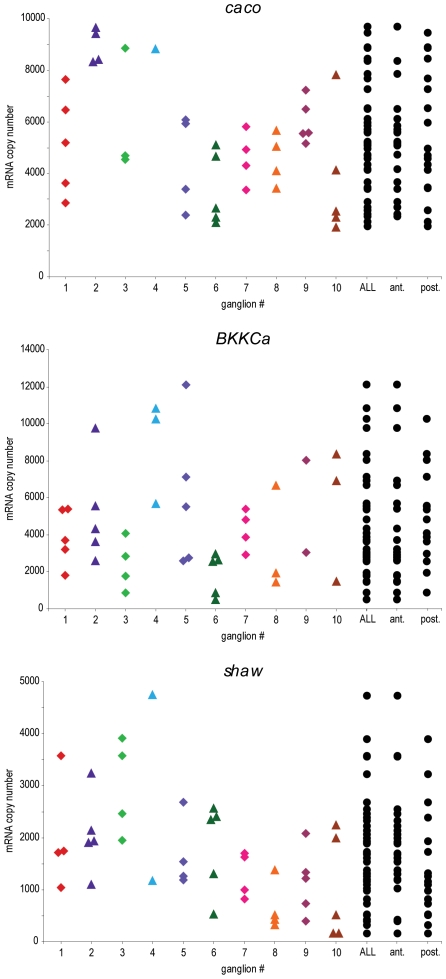
Ion channel mRNA measurements are not apparently more similar within an animal than across the population. For several example channels, measured in Group 1 LCs, there is no clear indication of clustering of ion channel measurements within an animal. There are no significant differences in channel mRNA levels between anterior (ant.) and posterior (post.) LCs (as determined by *t*-tests). Note that in ganglia where fewer than 5 LCs are shown, levels may artificially appear less variable.

In the STG, the electrically-coupled PD neurons have more similar ion channel mRNA and conductance measurements within an animal than PD neurons across different animals [12]. In the cardiac ganglion, the LCs did not appear to have more similar mRNA measurements within an animal than across the population (Fig. 4). Depending on the channel type, some ganglia are less variable, but there is little indication of a reproducible clustering phenomenon. No difference in ion channel measurements were seen between LCs found in the anterior portion of the ganglion compared to those found in the posterior region (*t*-test) (Fig. 4).

### Correlation of mRNA levels

**Figure 5:**
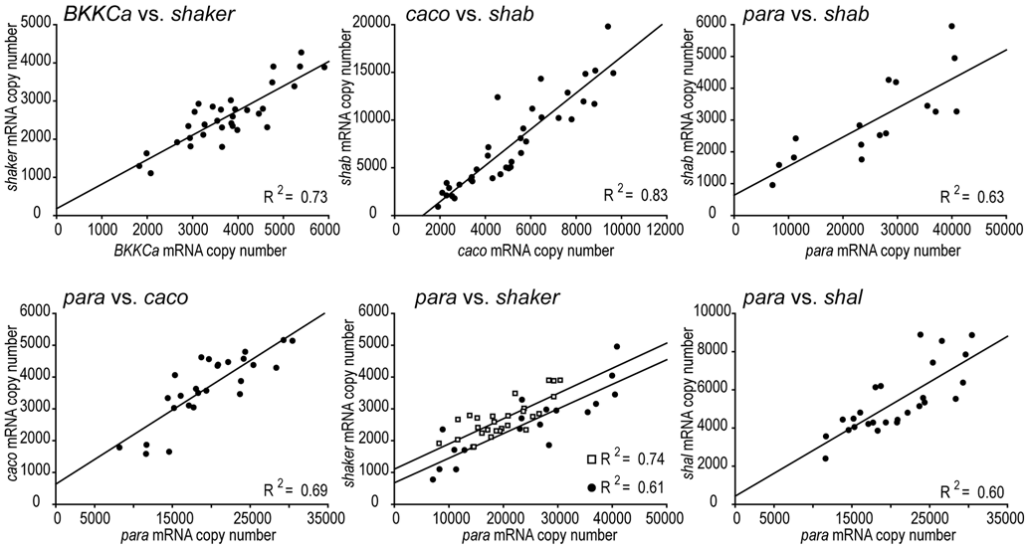
Levels of mRNA for 6 pairs of ion channels show significant strong (R^2^>0.6) positive correlations. *Top*: (left to right): *shaker* (I_A_) correlates with *BKKCa* (I_KCa_) in Group 3, *shab* (I_Kd_) correlates with *caco* (I_Ca_) in Group 1, *shab* correlates with *para* (I_Na_) in Group 2. *Bottom*: (left to right): *caco*, *shaker*, and *shal* (I_A_) correlate with *para* in Group 3. For *para* vs. *shaker*, data and correlations are shown also for Group 2 (white squares).

Although ion channel mRNA levels in crab STG neurons were variable within each cell type, there were strong correlations in mRNA levels in these neurons [6], [12]. Consequently, we looked for correlations in mRNA levels in LCs in the 28 possible pairs of channels. Positive correlations exist for 15 pairs of channels (R^2^ values: Table 1). Six pairs had strong correlations, with R^2^ values greater than, or equal to 0.60 (Fig. 5, Table 1) and nine pairs had R^2^ values between 0.28 and 0.58 (Table 1). The strongest correlation (R^2^ = 0.83) was between *shab* and *caco*. *BKKCa* and *shaker* were highly correlated (R^2^ = 0.73) and levels of *para* correlated with those of *shal* (I_A_; R^2^ = 0.6), *shab* (I_Kd_; R^2^ = 0.63), *caco* (I_Ca_; R^2^ = 0.69), and *shaker* (I_A_; Group 1 R^2^ = 0.61, Group 2 R^2^ = 0.74). Weak but statistically significant correlations were found between *BKKCa* and four transcripts: *IH* (Group 1 R^2^ = 0.40, Group 2 R^2^ = 0.36), *shal* (Group 1 R^2^ = 0.58, Group 3 R^2^ = 0.36), *caco* (Group 3 R^2^ = 0.50), and *para* (R^2^ = 0.40). *IH* expression was weakly correlated with *shal* (Group 3 R^2^ = 0.52) and *shaker* (R^2^ = 0.39), and *caco* was weakly correlated with *shal* (Group1 R^2^ = 0.28, Group 3 R^2^ = 0.46) and *shaker* (R^2^ = 0.44). Of the 7 pairs that were measured in two groups, 5 pairs showed similar results, but the weak correlations between *BKKCa* and *caco* and between *IH* and *shal* were significant in Group 3, but not in Group 1.

Because the reaction efficiency of the reverse transcription process can vary, causing false correlations between channels, channel mRNA levels for each cell were normalized to the cells' 18S rRNA levels, as described in Methods. To ensure that such normalization did not impose false correlations, we looked for correlations in unnormalized data. All statistically significant correlations in the normalized data were also significant in the unnormalized data, except for one pair: *para* and *IH* were correlated in the normalized data (R^2^ = 0.51), but not in the unnormalized data. As such, we refrain from declaring this pair as a set of correlated channels in LCs. As expected, the unnormalized data showed additional significant correlations, which may be due to variations in the efficiency of the reverse transcriptase or other processing variations.

## Discussion

Neurons must ensure that they always have in their membrane an appropriate balance of voltage-dependent conductances to produce their characteristic intrinsic activity, and to enable them to contribute to network function. Even in neuronal membranes with relatively few types of ion channels, such as the squid giant axon, the numbers of channels must be tightly controlled to maintain correct firing frequencies in response to synaptic drive. Most neurons, however, have many more than two or three types of channels. Consequently, these neurons confront a multi-dimensional balancing act to maintain their output patterns despite ongoing channel synthesis and turnover.

In principle, there are a number of strategies that a neuron could use to maintain an appropriate balance of ion channels: a) each channel gene could be regulated independently, according to some sensor of the number of that kind of channel in the membrane, b) the synthesis and turnover of all channels could be coordinately regulated so that an appropriate ratio was always maintained and matched to cell size, c) activity could be the controlled variable so that any balance of conductances that produces an acceptable activity pattern would be adequate, and d) some mixture of the above mechanisms. According to our data, some channels appear to be regulated in a coordinated fashion, however other channels may be controlled by a mixture of other strategies.

### The identity of the channels encoded by *shaker* and *caco* in *C. borealis*

We cloned a partial open reading frame of the *shaker* gene in *C. borealis*, which bears 97% amino acid identity to that in the closely related species, *Panulirus interruptus* (spiny lobster). Thus we expect that it corresponds to the same transient potassium current, I_A_ [36]. We also cloned a partial open reading frame of the *caco* gene that is similar to the *cac* gene in *Drosophila*, which encodes calcium channels homologous to vertebrate N-, P-, and Q-type channels [37], [38]. We currently lack biophysical evidence as to the characteristics of the channel encoded by *caco*. Because it is expressed in abundance, it likely corresponds to some part or the whole of the calcium current recorded in LCs. This current has two inactivation time constants and may be composed of two separate channels, or may be a single channel with both voltage- and calcium-dependent inactivation [28], [33], [39].

### Eight ion channel genes were quantified in cardiac ganglion LCs

This paper provides the first molecular profile of the ion channels that are transcribed and likely expressed in LCs. Despite over 10-fold variability in the absolute numbers of some transcripts, all LCs had generally similar profiles of the relative expression, e.g. *para* was the most abundant transcript in all cells measured, while *shaw* was generally the least abundant (Fig. 3). For several channels, however, mRNA copy number differed significantly between groups. Cells that were harvested in August (Group 1) had more *shal*, *IH*, *shab* and *caco* than those harvested in October and November (Groups 2 and 3). These differences suggest that some channels may undergo differential regulation, potentially related to the seasonal variations such as temperature, mating cycles or other environmental factors. Seasonal variations have been observed in the amplitude of I_A_ and I_h_ conductances in the STG neurons of *P. interruptus*, with the highest values recorded in spring, and the lowest in the winter months (Marie Goeritz, personal communication).

### Correlated expression of ion channels

In STG neurons, certain pairs of ion channel mRNA transcripts were linearly correlated [6], [12], however the pairs that were correlated differed between different cell types, suggestive that such correlations could be markers of cell identity or activity. The specific set of correlations found in the cardiac ganglion LCs (Table 1) differs from those found in the STG neurons. In the STG, the GM neuron is the only cell that, like the LCs, showed strong correlations between *para* and *shal* and between *para* and *shab* [12]. In GM, correlations between *BKKCa* and *para*, and *BKKCa* and *shal* were much stronger than in LCs. Additionally, GM showed several correlations that were absent in LCs, namely between *BKKCa* and *shab* and between *shab* and *shal*. The uniqueness of LCs correlations supports the idea that the correlations are unique to cell types.

In the STG [6], [12] and the cardiac ganglion LCs, all correlations thus far observed are positive correlations. To ensure that these positive correlations are not simply a function of cell size or variability in the processing, we normalized the data by a housekeeper gene, 18S, to remove this confound. The presence of only positive correlations in ion channel levels may be a feature of the yet unknown molecular mechanism that produces such correlations.

In LCs, there were significant correlations for fifteen pairs of conductances, six of these strong (R^2^>0.6) (Figure 5; Table 1), and nine weak (R^2^<0.6) (Table 1). The mechanisms that produce such correlations are unknown. Some channel genes may be transcribed in a correlated fashion, and post-transcriptional regulation would unequally alter transcript levels, thus weakening the measured correlation between them. If a channel is separately co-regulated with two different channels, some of its expression would correlate with one channel, but some of its expression would correlate with the other. This might also show up as a weak correlation between each pair of co-regulated channels.

Despite the fact that Group 1 cells expressed significantly more *shal*, *IH*, *shab* and *caco*, than the other groups, these cells exhibit similar sets of correlated ion channels. For example, although Group 1 *shal* was more than 50% greater than Group 3 *shal* (Fig. 2), both groups showed weak but significant correlations between *shal* and *caco* (Table 1). Additionally, both groups showed similar weak, but significant correlations in *BKKCa* and *IH* and *BKKCa* and *shal* (Table 1). However, two channel pairs, *BKKCa* and *caco*, and *IH* and *shal* were significantly correlated in Group 3, but not in Group 1. Because these correlations are weak, they may simply be on the threshold for being detected as significant, or these correlations (or one or more constituent channels) may be regulated depending on the season, or other environmental factors influencing animals from one group, but not another.

It is always a concern that the measured variability of ion channel expression could be the result of experimentally introduced error in mRNA measurements. The effect of random error would be to obscure any correlations we measure (Type II error), resulting in an underestimate of the strength or number of ion channel correlations. Measurement variability that results in all mRNA in a cell being under or over-reported would produce new correlations (Type I error). To avoid Type I errors, we normalized the data using a housekeeper gene (see Methods), and we present only those correlations that are significant in both normalized and unnormalized sets of data. Therefore, we are confident in our assessment that these are biologically meaningful correlations in mRNA abundance.

Although, for some genes, the electrically-coupled PD neurons in the STG show less variability within an animal than across the population, the electrically-coupled LCs do not appear to be more similar within an animal (Fig. 4). The cause of this difference between networks is not obvious. Within each network, both LCs and PDs are electrically-coupled, share similar activity, similar neuromodulatory influences, and are driven by electrical-coupling to pacemaker neurons. Perhaps the strength of the electrical coupling between cells, or other influences on the PD neurons cause the intra-animal similarities.

### Functional significance of ion channel correlations

Our results suggest that, while neurons may have variable amounts of ion channel mRNA, correlations exist between channel transcripts that may aid in maintaining neuronal function. Among the 14 significantly correlated channel pairs, all but three involve an inward current and an outward current. A balance between inward and outward currents may help to maintain the membrane potential in a physiologically functional range. Among the strongly correlated pairs, *para* is correlated with 3 outward currents. Such a correlation could help to maintain action potential shape and frequency. A balance between I_Ca_ and I_K_, suggested by the correlations between *caco* and *shab* and between *caco* and *BKKCa* may be important for the rhythmic activity of the cardiac ganglion network. In isolated LCs in the crab *Portunus sanguinolentus*, a balance between I_Ca_ and the outward currents is thought to underlie the transient depolarization, repolarization, and interburst intervals that may shape LC activity in the network [28].

These data are consistent with the hypothesis that variability in channel expression may reflect a homeostatic process by which the neuron “tunes” its own conductances to maintain a target activity [3], [6]. Furthermore, correlations between conductances may represent a mechanism by which certain ratios that can maintain target activity can be enforced [12], [40]. For example, in cultured *Drosophila* neurons, long-term removal of I_Ca_ causes a homeostatic decrease in I_KCa_ and increase in I_A_, but does not appear to involve changes in channel kinetic properties, as would be expected by post-translational modifications of channels [33]. Further, a direct functional role for correlated conductances has been previously demonstrated in STG neurons of the spiny lobster, where I_h_ and I_A_, conductances with opposing effects on activity, were found to occur with a set ratio in their amplitudes [40]. By overexpressing *shal*, MacLean et al. [40], [41] increased I_A_, but found that I_h_ had increased commensurately, resulting in surprisingly small changes in cell activity. Using a conductance-based model [40], the authors demonstrated that increasing I_A_ or I_h_ alone would change the activity of the neuron, but increasing both together maintained neural activity. Such regulation of cellular output is one potential mechanism for generating the correlations in ion channel gene expression measured in the STG, and now in the cardiac ganglion LCs.

Although ion channel correlations may be helpful in maintaining neural activity, they do not appear to be required to do so. Taylor et al. [10] investigated the functional significance of ion channel correlations in the lateral pyloric (LP) neuron of the crab STG using a multi-compartment conductance-based model. They randomly generated models that were screened for LP-like activity and properties, resulting in ∼1300 models. This population of models did not have conductance correlations corresponding to the correlations in ion channel mRNA that were measured in LP neurons [12]. These results suggest that the correlations may not be required to produce the phenotypic firing patterns of the LP neuron, but may be present for developmental regulation [10]. The correlations may, therefore, be an epiphenomenon resulting from the mechanism of channel transcription. However it is also possible that, while channel correlations may not be required to maintain activity, they could be useful. Neuronal activity may be maintained by a preserving a direct correlation between ion channels, but it may also be possible to maintain activity in other ways, such as altering three or four other conductances to balance the effects of changing one.

## Materials and Methods

### Cloning of ion channel genes from *C. borealis*

The methods for cloning are as described previously [6]. Briefly, channels were cloned from a cDNA template derived from mixed nervous system tissue from *C. borealis*. We used degenerate primer pairs based on conserved amino acid sequence compared across multiple species. PCR products of predicted length were cloned into the pGem-T easy plasmid vector (Promega) and sequenced using dye terminator cycle sequencing (DTCS Quick Start Kit, Beckman Coulter). Sequences obtained were compared to orthologous sequences using BlastX (NCBI). Accession numbers for these new sequences are as follows: (Cab = *Cancer borealis*) Cab-*shaker* FJ263946; Cab-*cacophony* FJ263945.

### Harvesting neurons

*C. borealis* were purchased from Commercial Lobster (Boston, MA) and maintained in recirculating artificial seawater tanks at 10–13°C for 1 day to several weeks. Before dissection, animals were anesthetized by immersion in ice for 30 mins. All dissections were carried out in chilled physiological saline (composition in mmol: NaCl, 440; KCl, 13; MgCl_2_, 26; CaCl_2_, 13, Trizma base, 11; maleic acid, 5; pH 7.45). Heart and cardiac ganglion dissections were performed as previously described [42]. Briefly, hearts were dissected from crabs and pinned ventral side up in saline-filled Petri dishes lined with Sylgard (Dow Corning, Midland, MI). The ventral wall of each heart was cut medially and the interior of the heart chamber exposed to reveal the cardiac ganglion along the dorsal wall. The cardiac ganglion was removed from the heart tissue, pinned in saline-filled Petri dishes lined with Sylgard. Ganglia were desheathed around the area where large cells (LCs) were visually identified, and a Vaseline well was built around each ganglion. To enable removal of the neurons, the ganglia were exposed to protease (Sigma, St. Louis, MO) for several minutes. The protease was washed off with several saline washes, and the well was filled with a cold (∼−10°C–0°C) solution of 70% ethylene glycol and 30% saline. This solution was exchanged several times to ensure purity of concentration. The ganglia were put in a −80°C freezer for 1–2 hours. The cells were manually removed with fine forceps and each cell was placed in a cryogenic tube with 350 ml lysis buffer (buffer RLT, Qiagen, Valencia, CA) and 1% β-mercaptoethanol. The tubes were immediately placed on ice, then frozen in a −80°C freezer within 10 minutes of adding the cells. The neurons were harvested at three separate times during a 15 month period. Group 1 was harvested during the month of August, and the following year Group 2 was harvested in October, and Group 3 in November. Some results differ between groups and are therefore presented according to their group (not pooled).

### Quantitative single-cell RT-PCR

Quantitative RT PCR was performed as in [6]. Except where specified, the raw mRNA copy numbers for each channel from each cell were normalized to control for variations in reverse transcriptase efficiency. We measured the critical threshold (Ct) for 18S rRNA (proportional to its copy number) in each cell and calculated a population mean for each batch. For each cell, we calculated a scale factor as a ratio of the cell's 18S level to the mean 18S level of its batch, representing the relative size of each cell with respect to the mean cell size. We normalized each transcript level by multiplying raw transcript levels by the scale factor (as in [6], [11]). Primers specific for real–time PCR detection of *shal, BKKCa, shab, shaw*, and *IH* and 18S rRNA using Sybr Green were developed and designed using Primer3 software and are the same as previously reported [6], [12]. New primers also were generated for the quantification of *shaker*, *cacophony*, and *para* as follows: Shaker–F 5′– TTTATCAAGGAGGAGGAGCG –3′; Shaker–R 5′ –ATGATGGCCACCACTCTAGC– 3′ ; Cacophony–F 5′– GTATCCGGCGGACAGTAAAG –3′; Cacophony–R 5′– AAACTTGGTGAGAAATGGCG –3′; Para–F 5′– ATCTTTGCCATCATGGGTGT –3′; Para–R 5′– AAACACATTAGGCGGTCTGG –3′.

### Electrophysiological recordings

Heart and cardiac ganglion dissections were performed as previously described [42], and summarized above. Electrophysiological recordings were taken as previously described [32]. Briefly, the ganglion was pinned in a sylgard-lined dish and perfused with physiological saline. Intra-axonal recordings were made using 20–40 MΩ glass microelectrodes filled with 0.6 mol l^−1^ K_2_SO_4_ and 20 mmol l^−1^ KCl and an Axoclamp 2A (Axon Instruments). Extracellular recordings were made by placing stainless steel pin electrodes inside vaseline wells that encompass a section of the cardiac ganglion trunk.

### Statistical analyses

Data were analyzed using Systat v11 software (Systat Software Inc., San Jose, CA). Significant correlations among pairs of channels were based on Pearson's product-moment correlation coefficients. Differences between mean levels of expression of channels between different groups were analyzed with *t*-tests. All regression plots were created using SigmaPlot v10 (Systat Software Inc) software.

### Outlier analysis

Because we employed SybrGreen for the realtime analysis, a melt curve was generated for each sample. Any samples that revealed multiple amplification products were excluded from the analysis. For the resulting data set, univariate outlier analysis was conducted for each batch by converting the individual values in the data set into their corresponding standard z-scores according to the formula z_i_  =  (X_i_ – M)/SD where M is the mean and SD is the inferential standard deviation. A value was considered an outlier if its z-score was ±2.5 or beyond. This resulted in the removal of 4 of 276 samples from Group 1, 3 of 96 samples from Group 2, and 1 of 210 samples from Group 3.

## References

[1] Achard P, De Schutter E (2006). Complex parameter landscape for a complex neuron model.. PLoS Computational Biology.

[2] Golowasch J, Goldman MS, Abbott LF, Marder E (2002). Failure of averaging in the construction of a conductance-based neuron model.. J Neurophysiol.

[3] Marder E, Goaillard JM (2006). Variability, compensation and homeostasis in neuron and network function.. Nat Rev Neurosci.

[4] Prinz AA, Billimoria CP, Marder E (2003). Alternative to hand-tuning conductance-based models: construction and analysis of databases of model neurons.. J Neurophysiol.

[5] Prinz AA, Bucher D, Marder E (2004). Similar network activity from disparate circuit parameters.. Nat Neurosci.

[6] Schulz DJ, Goaillard JM, Marder E (2006). Variable channel expression in identified single and electrically coupled neurons in different animals.. Nat Neurosci.

[7] Tobin AE, Calabrese RL (2006). Endogenous and half-center bursting in morphologically-inspired models of leech heart interneurons.. J Neurophysiol.

[8] Swensen AM, Bean BP (2005). Robustness of burst firing in dissociated purkinje neurons with acute or long-term reductions in sodium conductance.. J Neurosci.

[9] Taylor AL, Hickey TJ, Prinz AA, Marder E (2006). Structure and visualization of high-dimensional conductance spaces.. J Neurophysiol.

[10] Taylor AL, Goaillard JM, Marder E (2009). How multiple conductances determine electrophysiological properties in a multicompartment model.. J Neurosci.

[11] Liss B, Franz O, Sewing S, Bruns R, Neuhoff H (2001). Tuning pacemaker frequency of individual dopaminergic neurons by Kv4.3L and KChip3.1 transcription.. Embo J.

[12] Schulz DJ, Goaillard J-M, Marder EE (2007). Quantitative expression profiling of identified neurons reveals cell-specific constraints on highly variable levels of gene expression.. Proceedings of the National Academy of Sciences.

[13] Desai NS, Rutherford LC, Turrigiano G (1999). Plasticity in the intrinsic excitability of cortical pyramidal neurons.. Nat Neurosci.

[14] Golowasch J, Abbott LF, Marder E (1999). Activity-dependent regulation of potassium currents in an identified neuron of the stomatogastric ganglion of the crab *Cancer borealis*.. J Neurosci.

[15] LeMasson G, Marder E, Abbott LF (1993). Activity-dependent regulation of conductances in model neurons.. Science.

[16] Spitzer NC (1999). New dimensions of neuronal plasticity.. Nat Neurosci.

[17] Turrigiano G, Abbott LF, Marder E (1994). Activity-dependent changes in the intrinsic properties of cultured neurons.. Science.

[18] Turrigiano G, LeMasson G, Marder E (1995). Selective regulation of current densities underlies spontaneous changes in the activity of cultured neurons.. J Neurosci.

[19] Turrigiano GG (1999). Homeostatic plasticity in neuronal networks: the more things change, the more they stay the same.. Trends in Neurosciences.

[20] Turrigiano GG, Leslie KR, Desai NS, Rutherford LC, Nelson SB (1998). Activity-dependent scaling of quantal amplitude in neocortical neurons.. Nature.

[21] Lammel S, Hetzel A, Häckel O, Jones I, Liss B (2008). Unique Properties of Mesoprefrontal Neurons within a Dual Mesocorticolimbic Dopamine System.. Neuron.

[22] Hartline DK (1967). Impulse identification and axon mapping of the nine neurons in the cardiac ganglion of the lobster *Homarus americanus*.. J Exp Biol.

[23] Hartline DK (1979). Integrative neurophysiology of the lobster cardiac ganglion.. Amer Zool.

[24] Mayeri E (1973). Functional organization of the cardiac ganglion of the lobster, *Homarus americanus*.. J Gen Physiol.

[25] Maynard DM (1955). Activity in a crustacean ganglion. II Pattern and interaction in burst formation.. Biol Bull.

[26] Tazaki K, Cooke IM (1979a). Spontaneous electrical activity and interaction of large and small cells in cardiac ganglion of the crab, *Portunus sanguinolentus*.. J Neurophysiol.

[27] Tazaki K, Cooke IM (1990). Characterization of Ca current underlying burst formation in lobster cardiac ganglion motorneurons.. J Neurophysiol.

[28] Tazaki K, Cooke IM (1986). Currents under voltage clamp of burst-forming neurons of the cardiac ganglion of the lobster (*Homarus americanus*).. J Neurophysiol.

[29] Tazaki K, Cooke IM (1979c). Ionic bases of slow, depolarizing responses of cardiac ganglion neurons in the crab, *Portunus sanguinolentus*.. J Neurophysiol.

[30] Tazaki K, Cooke IM (1979b). Isolation and characterization of slow, depolarizing responses of cardiac ganglion neurons in the crab, *Portunus sanguinolentus*.. J Neurophysiol.

[31] Tazaki K, Cooke IM (1983). Neuronal mechanisms underlying rhythmic bursts in crustacean cardiac ganglia.. Symp Soc Exp Biol.

[32] Cruz-Bermúdez ND, Marder E (2007). Multiple modulators act on the cardiac ganglion of the crab, *Cancer borealis*.. J Exp Biol.

[33] Peng IF, Wu C-F (2007). *Drosophila* cacophony channels: A major mediator of neuronal Ca^2+^ currents and a trigger for K^+^ channel homeostatic regulation.. J Neurosci.

[34] Kim M, Baro DJ, Lanning CC, Doshi M, Farnham J (1997). Alternative splicing in the pore-forming region of shaker potassium channels.. J Neurosci.

[35] Kim M, Baro DJ, Lanning CC, Doshi M, Moskowitz HS (1998). Expression of Panulirus shaker potassium channel splice variants.. Receptors Channels.

[36] Baro DJ, Levini RM, Kim MT, Willms AR, Lanning CC (1997). Quantitative single-cell-reverse transcription-PCR demonstrates that A-current magnitude varies as a linear function of *shal* gene expression in identified stomatogastric neurons.. J Neurosci.

[37] Rieckhof GE, Yoshihara M, Guan Z, Littleton JT (2003). Presynaptic N-type calcium channels regulate synaptic growth.. J Biol Chem.

[38] Smith LA, Wang X, Peixoto AA, Neumann EK, Hall LM (1996). A *Drosophila* calcium channel alpha 1 subunit gene maps to a genetic locus associated with behavioral and visual defects.. J Neurosci.

[39] Cooke IM (2002). Reliable, responsive pacemaking and pattern generation with minimal cell numbers: the crustacean cardiac ganglion.. Biol Bull.

[40] MacLean JN, Zhang Y, Goeritz ML, Casey R, Oliva R (2005). Activity-independent coregulation of I_A_ and I_h_ in rhythmically active neurons.. J Neurophysiol.

[41] MacLean JN, Zhang Y, Johnson BR, Harris-Warrick RM (2003). Activity-independent homeostasis in rhythmically active neurons.. Neuron.

[42] Cruz-Bermúdez ND, Fu Q, Kutz-Naber KK, Christie A, Li L (2006). Mass spectrometric characterization and physiological actions of GAHKNYLRFamide, a novel FMRFamide-like peptide from crabs of the genus *Cancer*.. J Neurochem.

